# Sympathetic Nerve Activity and Blood Pressure Response to Exercise in Peripheral Artery Disease: From Molecular Mechanisms, Human Studies, to Intervention Strategy Development

**DOI:** 10.3390/ijms231810622

**Published:** 2022-09-13

**Authors:** Lu Qin, Jian Cui, Jianhua Li

**Affiliations:** Heart & Vascular Institute, The Penn State University College of Medicine, Hershey, PA 17033, USA

**Keywords:** sympathetic nerve activity, arterial blood pressure, peripheral artery disease, static exercise, muscle afferent nerve, heat treatment

## Abstract

Sympathetic nerve activity (SNA) regulates the contraction of vascular smooth muscle and leads to a change in arterial blood pressure (BP). It was observed that SNA, vascular contractility, and BP are heightened in patients with peripheral artery disease (PAD) during exercise. The exercise pressor reflex (EPR), a neural mechanism responsible for BP response to activation of muscle afferent nerve, is a determinant of the exaggerated exercise-induced BP rise in PAD. Based on recent results obtained from a series of studies in PAD patients and a rat model of PAD, this review will shed light on SNA-driven BP response and the underlying mechanisms by which receptors and molecular mediators in muscle afferent nerves mediate the abnormalities in autonomic activities of PAD. Intervention strategies, particularly non-pharmacological strategies, improving the deleterious exercise-induced SNA and BP in PAD, and enhancing tolerance and performance during exercise will also be discussed.

## 1. Introduction

Peripheral artery disease (PAD) is a common and disabling cardiovascular disease that affects over 200 million worldwide and ~20% of Americans over age 60 [1,2,3,4]. Patients with PAD are at a high risk of myocardial infarctions, cerebral vascular accidents, and all-cause mortality with a death rate like that in patients with coronary or cerebral vascular disease [5,6,7]. The atherosclerotic alternation in the affected vessel results in progressive narrowing of the lower extremity conduit vasculature and eventually leads to severe limb ischemia. As one of the consequences of limb ischemia, the syndrome of “intermittent claudication” in PAD patients which is characterized by pain in the lower limbs that occurs with walking and is relieved by rest limits their tolerance and performance in daily physical activities.

Compared with the major advances seen in the management of other cardiovascular diseases such as coronary artery disease and systolic heart failure, therapeutic options other than surgery for PAD remain extremely limited [8]. Several pharmacological interventions have been evaluated for use in patients with claudication symptoms, but efficacy has only been reported for cilostazol and anti-platelet agents [9,10]. In fact, exercise training (advice to walk more often) is commonly recommended for PAD patients. It has been supported by studies [11,12,13] that supervised treadmill exercise is effective in attenuating pain perception and improving the exercise performance of PAD patients. However, the implementation of exercise into the daily lives of PAD patients is met with significant challenges. During exercise, sympathetic nerve activity (SNA) and blood pressure (BP) responses are amplified in PAD patients [14,15,16,17], which is associated with a higher risk and incidence of cardiovascular events [18,19].

In this regard, experimental animal models are necessary for studying the underlying molecular mechanisms leading to the exaggerated SNA and BP responses in the pathological conditions of PAD; human clinical studies in both the healthy population and PAD patients are essential to examine the clinical conditions and validate the results of the mechanism studies with approved medications. More importantly, by incorporating the animal study and human study, intervention studies on the treatment targets are vital to develop strategies aiming to be effective and low-cost and to attenuate the above adverse conditions in PAD patients.

## 2. Sympathetic Nerves and BP Regulation during Exercise in PAD

### 2.1. Exercise Pressor Reflex (EPR)

**Figure 1:**
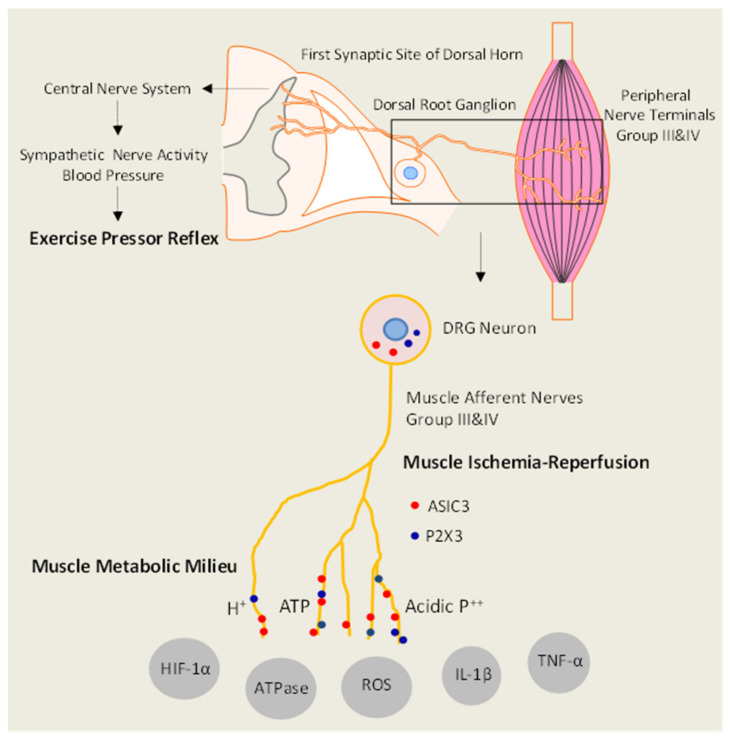
Diagram indicates the potential signaling pathways leading to the exaggerated EPR in IR rats via enhancing acidic metabolites, ATP, and proinflammatory cytokines (PICs) in skeletal muscle and thereby stimulating ASIC3 and P2X3 receptors in muscle sensory nerves. This proposal will examine the integrated signals in both skeletal muscle and primary sensory neurons involved in the EPR of rats with femoral artery occlusion followed by reperfusion. ASIC3: acid-sensing ion channel 3; P2X3: purinergic P2X subtype 3; HIF-1α: hypoxia-induced factor-1α; ROS: reactive oxygen species; IL-1β: interleukin-1β; TNF-α: tumor necrosis factor-α.

During the muscle movements of exercise, the sympathetic nervous activity (SNA) increases, resulting in increased arterial BP and heart rate (HR), myocardial contractility, and peripheral vasoconstriction [20,21]. Two mechanisms: central command and exercise pressor reflex (EPR) [21,22,23,24,25,26] are considered involved in this regulatory process. Specifically, “Central Command” [27] is initiated by a volitional signal emanating from central motor units and then induces the enhancement of SNA; and the “Exercise Pressor Reflex” [26,28] originates from the signal inputs from the afferents of the contracting skeletal muscle and then induces a subsequent autonomic reflex. For the specific types of signal input, the EPR responds to metabolic stimulation (i.e., “metaboreceptor” stimulation in Group IV afferents) and mechanical deformation (i.e., “mechanoreceptor stimulation” in Group III afferents) in the muscle afferents receptive field [29]. Thin fiber muscle afferent nerves are engaged following the stimulation of the receptors during the exercise, therefore inducing the activation of cardiovascular nuclei in the brainstem [28]. Figure 1 illustrates the activation of the EPR and its neural pathways.

### 2.2. EPR in PAD Patients

**Figure 2:**
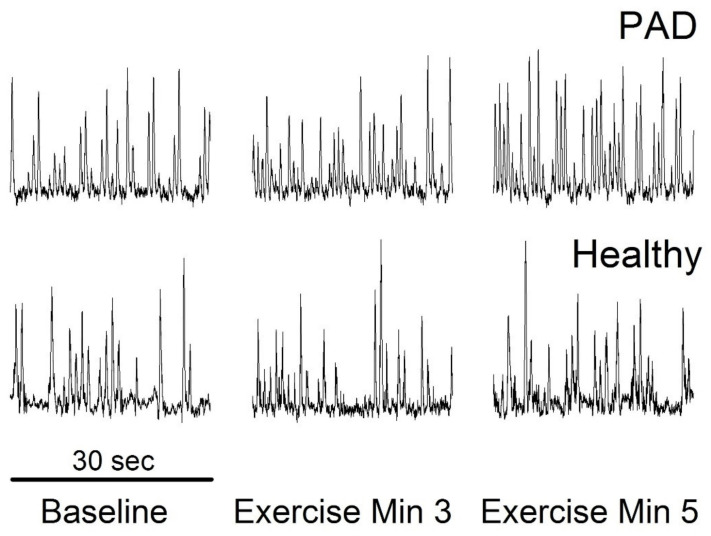
MSNA during plantar flexion with incremental loading (2 kg, +1 kg/min) in a PAD patient. The exercise was ended after min 5 due to the tolerance level of this patient. In the matched healthy control subject, increases in MSNA and BP occurred after min 7 with a 9 kg workload. (Abstract presented at EB 2021; unpublished figure).

In PAD patients, the pressor response to walking is significantly greater than that in healthy control subjects [14,15,16,17]. Human studies further indicate that the responses in BP, renal vasoconstriction, and total peripheral resistance (TPR) during plantar flexion exercise are accentuated in PAD [30,31]. In these studies, the increase in BP occurred before the subjects reported pain. Thus, it is believed that an exaggerated EPR is a major determinant of why BP rises with exercise in PAD [32]. The muscle SNA (MSNA) increases in PAD occurred early and were much greater than those at the same exercise time/workload in matched healthy control subjects [33]. Figure 2 shows increased MSNA in PAD patients. It is believed that an exaggerated MSNA response contributes to the accentuated TPR and BP responses to leg exercise in PAD patients and any mechanisms for intervention/therapy decreasing the MSNA response to exercise would alleviate the exaggerated EPR in PAD. 

## 3. Experimental Models to Study the Pathological Status in Human PAD

### 3.1. Blood Flow Restriction (BFR) and Ischemia-Reperfusion (IR) in Healthy Subject

Other than involving PAD patients, there are two popularized experimental models to simulate PAD, namely BFR and IR, in healthy humans. They provide low-risk and feasible ways to mimic the pathological status (e.g., ischemia and ischemia-reperfusion) in PAD. 

BFR: Regarding the pathophysiology of PAD, the consequences of limb ischemia have been emphasized [34,35,36,37,38]. It is known that the EPR is amplified as oxygen delivery to skeletal muscle is reduced [39]. Acute flow limitation during exercise also raises BP in a canine hindlimb occlusion model [40]. In humans, BFR is achieved by placing a pressure cuff proximal to the working muscle and inflating it to achieve a pressure-limiting flow to the muscles. BFR has been used for augmenting the peripheral adaptations to resistance training [41,42,43,44,45,46]. Importantly, a recent report [47] and data showed that the BP response to exercise is accentuated under BFR conditions, even when the blood flow is not fully occluded. Thus, there is technical and ethical feasibility of using the BFR model in healthy humans to simulate the flow limitation in PAD. 

**Figure 3:**
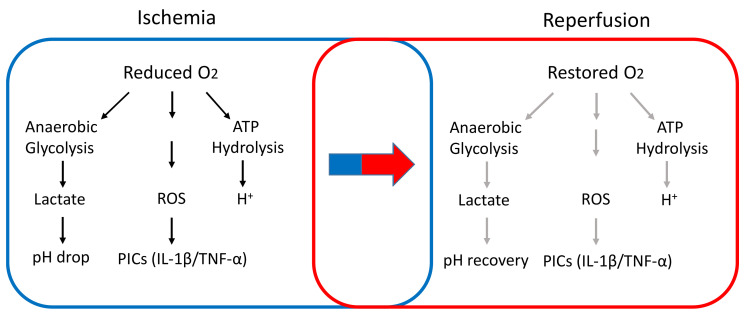
A diagram illustrates the potential effects of IR on muscle metabolic milieu in the hindlimb. We will study integration of the signaling pathways between skeletal muscle metabolites and primary muscle sensory neurons in regulation of the exercise pressor reflex in IR rats. ROS: reactive oxygen species; PIC: proinflammatory cytokine. Dark arrow indicates “increase” and light arrow indicates “alleviate”. Note that the diagram is simplified to show the mechanisms more related to our proposed studies, but not all the molecular mediators responsible for IR are shown.

IR: The IR injury is a main feature of various cardiovascular diseases, including myocardial infarction, stroke, and PAD [48,49]. Figure 3 illustrates the potential effect of IR on the metabolic milieu in the skeletal muscle tissue. The tissue damage associated with ischemic events occurs due to a combination of ischemia and paradoxical reperfusion following the restoration of blood flow to ischemic tissue, commonly referred to as IR injury. Following ischemia, the muscles may be salvaged by reperfusion. However, the re-introduction of oxygen to hypoxic muscles can also lead to damage by oxygen-derived free radicals. In PAD patients, IR injury was observed after limb revascularization [50,51]. Moreover, walking in PAD patients can induce ischemia (indicated with pain), while reperfusion can occur after stopping walking. Thus, intermittent claudication has been linked to ischemia followed by reperfusion leading to repeated IR injuries in their daily life [35,48,49,52].

Prior studies [53,54,55] have employed a 20-min period of ischemia followed by a 20-min period of reperfusion (i.e., 20–20 min) to induce IR stress in a limb of healthy subjects to examine the effects of IR stress on vascular function [56,57]. This model has been used to simulate the IR stress in PAD and examine the effects of IR on the EPR. In our prior study [58], subjects performed fatiguing handgrip exercise before and ~20 min after a 20-min period of muscle ischemia (i.e., the limb circulation was totally occluded) in the control trial. The results showed that the MSNA responses to handgrip were accentuated after the 20-min period of muscle ischemia. Thus, the IR stress in healthy subjects and PAD patients with leg revascularization can be used to examine the role played by the IR in regulating the EPR.

### 3.2. Animal Models of Studying Human PAD

By effectively restricting and eliminating the blood flow in the affected limbs, the animal model of PAD proves an essential tool for studying the underlying molecular mechanism under the ischemic-related etiological and pathological conditions in PAD patients. In a recent review of the literature, we summarized the features of representative animal models of PAD [59]. Based on different feasibilities and approaches, the experimental animal options are rodents (e.g., mice, rats, and rabbits) and large animals such as swine. Methods of inducing blood flow restriction and elimination include single/double occlusion with or without blocking the branches in the femoral artery [60,61,62,63,64,65], ultra-sound assisted endovascular occlusion [66,67], and chemical-induced thrombus ischemia [68], etc. In the following sections, we will focus on discussing two animal models that mimic the blood flow restriction and ischemia-reperfusion status in human PAD: femoral artery occlusion/ligation and hindlimb ischemia-reperfusion.

#### 3.2.1. Femoral Artery Occlusion/Ligation

**Figure 4:**
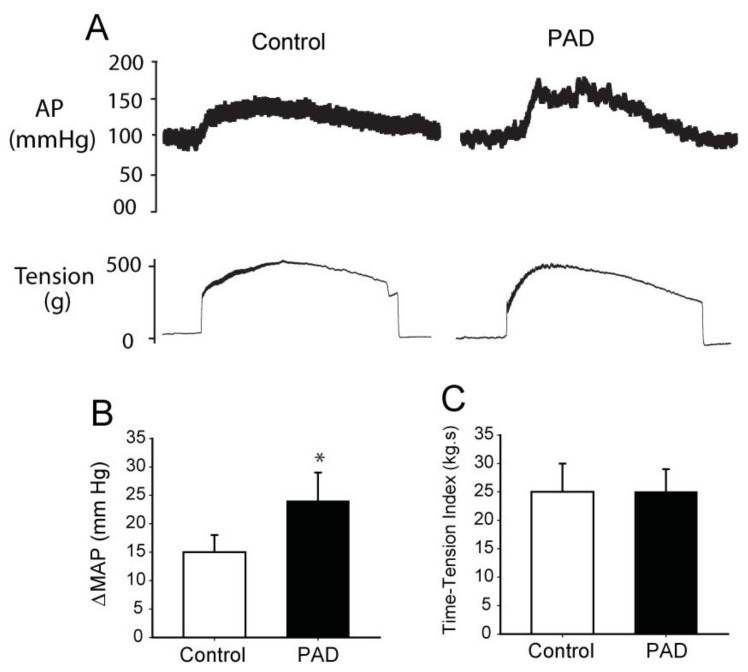
Mean arterial pressure (AP) response to static muscle contraction (30 s) induced by electrical stimulation of the L4&5 ventral roots in controls rat and PAD rats. (**A**) Typical recordings of arterial pressure (AP) response to static muscle contraction in control and PAD rats. (**B**,**C**) A greater MAP response was seen in PAD rats than in control rats, without different muscle tension between two groups * *p* < 0.05 between control and PAD.

Femoral artery occlusion in rats has been widely used to study human PAD [69] as it mimics one of the critical characteristics seen in PAD patients, namely intermittent claudication manifested by insufficient blood flow to the legs during exercise or slightly decreased blood flow to the legs under resting conditions. Notably, 24 h or 72 h femoral occlusion exaggerates BP response to muscle contraction (Figure 4 and muscle metabolites (e.g., acidic products and ATP) in the occluded limb (Figure 1), but not in the opposing control limb of the same rats [70,71]. Meanwhile, it has also been reported that the BP response during exercise is still exaggerated ~1 month and ~2 months after the femoral artery occlusion [72]. These findings parallel those reported in humans, showing that the BP response to walking is enhanced in PAD and the BP response during the exercise with the “diseased” limb is greater than that during the exercise with the “non-diseased” limb [16]. Therefore, it is indicated that a rat model of the femoral artery ligation is suitable for studying exercise-induced ischemia that occurs in PAD. Moreover, PAD in human subjects is not solely a disease of large vessel obstruction, but it is a disease of large vessel obstruction in the setting of a chronic disease process (atherosclerosis) that is influenced by oxidative stress and inflammation [73]. Femoral artery occlusion increases products of oxidative stress in the hindlimb muscles of rats and activates inflammatory signals (i.e., IL-6 and TNF-α) [74,75,76] as shown in Figure 1. This also makes the femoral occlusion model reflective of human conditions. 

#### 3.2.2. Hindlimb Ischemia-Reperfusion

The hindlimb IR is induced by femoral artery ligation, followed by re-opening the ligation and is used to study PAD. To date, little is known about the engagement of a reperfusion component of IR injury in the pathophysiological processes of BP response in PAD. In the previous studies with forelimb IR models with mice, 18 h of blood flow reperfusion following 6 h of ischemia lowered the pain threshold of the affected limbs and increased the BP response during the dynamic global exercise. Meanwhile, the mRNA levels of primary sensory receptors (e.g., acid-sensing ion channel 3, ASIC3 and purinergic P2X3, P2X3) and the receptors for cytokines (e.g., interlukin-1β receptor, IL-1 βr) in the DRG were also increased [77]. With further development of the hindlimb IR model, we characterized a rat model of IR, showing that BP response to muscle contraction in different time courses following IR (e.g., 18, 66, and 114 h) were exaggerated. Notably, the increment of BP response 18 h following reperfusion was the most profound. It should also be noted that the BP response to muscle contraction was evaluated in decerebrated animals, which excluded the effect of central command. Further underlying mechanism studies with this IR model have shown that the intra-arterial injection of lactic acid (activator of ASIC3 receptor) and α,β me-ATP (activator of P2X3 receptor) were amplified in rats (IR18h) who experienced 6 h of femoral artery ligation followed by 18 h of reperfusion [78]. The increasing levels in BP response were similar in IR18h rats and rats with 24 h of femoral artery occlusion (24 h-occlusion rats). Moreover, the protein levels of ASIC3 and P2X3 expression in dorsal root ganglion (DRG) were increased to a similar degree in IR18h rats and 24 h-occlusion rats. These data suggest that reperfusion following 6 h ischemia is likely a factor leading to the remaining exaggeration of the BP response in IR rats and it is rational to utilize a rat model of IR18h for studying IR injury in PAD. 

## 4. Molecular Mechanisms Leading to Exaggerated SNA and BP Responses in PAD

### 4.1. Effects of Muscle Metabolic Products and Their Responsive Receptors (Figure 5 and Figure 6)

Using this PAD rat model with femoral artery ligation/occlusion, the previous studies have demonstrated that the SNA and pressor responses to muscle contraction and stimulation of muscle metabolite receptors i.e., acid-sensing ion channel 3 (ASIC3), purinergic P2X (subtype P2X3), transient receptor potential vanilloid 1 and ankyrin 1 (TRPV1 and TRPA1) are amplified in PAD rats as compared with control rats [71,79,80,81,82,83]. In addition, other receptors in muscle afferent nerves including µ-opioid/δ-opioid, bradykinin (BK) B2, prostaglandin (PGE2) EP4, and thromboxane (TP) receptors are engaged in the reflex responses in processing chronic ischemia of the hindlimb muscles [71,84,85,86,87]. 

In addition to the previous works on the roles of ASICs, TRPV1, and P2X receptors [70], we will focus on the updated research of ASIC3 and the interaction effect of ASIC3 and P2X3 on muscle sensory nerves in mediating the exaggerated sympathetic response in hindlimb muscle ischemia seen in PAD patients. Those receptors to be studied are expressed at both the peripheral terminals and the cell body of the sensory afferent neurons-DRG. With greater feasibility, receptor activity of DRG cell bodies has been used frequently as a surrogate for the nerve-ending receptor activity and physiology [88,89]. In particular, the whole cell patch-clamp methods are used to characterize the precise mechanisms by which those receptors mediate responses in the DRG neurons [88,89].

**Figure 5:**
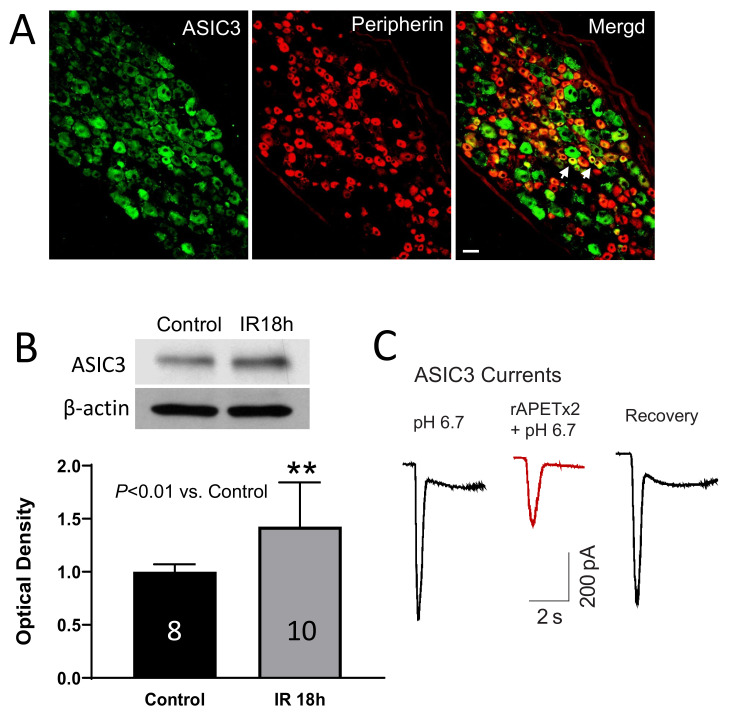
(**A**) The dual immunofluorescence method showing co-existence of ASIC3 and peripherin staining in DRG neurons. Arrows indicate representative cells positive for both ASIC3 and peripherin after they were merged. Scale bar = 50 µm. Peripherin was used to label C-fiber of DRG neurons. (**B**) Bands and averaged data (mean ± SD) showing that IR increased the protein levels of ASIC3 in DRGs. ** *p* < 0.01 between control and IR 18h rats. (**C**) Original traces of patch clamp showing that the amplitudes of ASIC current (elicited by pH 6.7 solution) in were largely decreased after application of ASIC3 antagonist rAPETx2 (1 µM).

**Figure 6:**
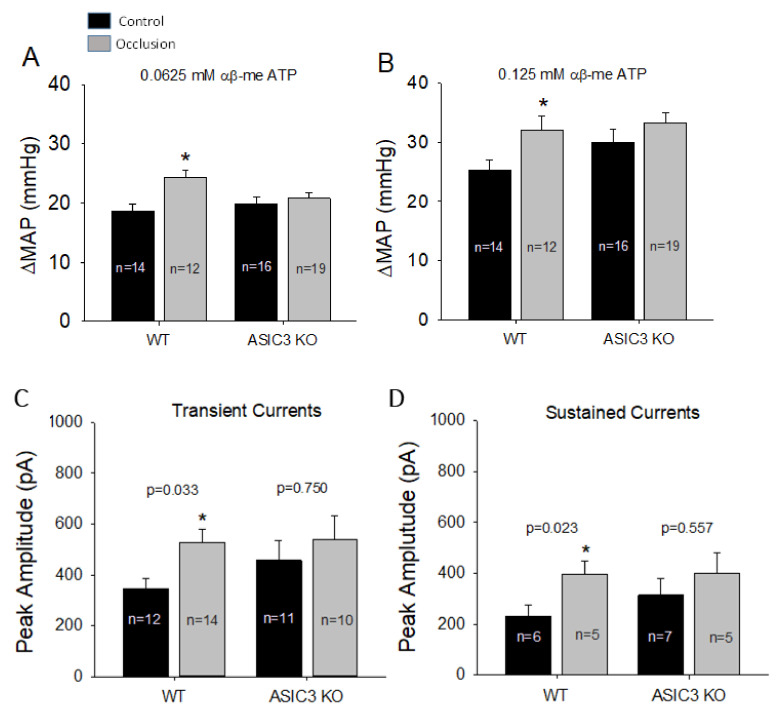
Effect of ASIC3 KO on BP response and DRG currents to activation of P2X3. (**A**,**B**): Two doses of αβ-me ATP were given intra-arterially into the hindlimb muscles to stimulate P2X3 in muscle afferents; and activation of P2X3 receptors amplified MAP response to a greater degree in WT rats with femoral occlusion, but not in ASIC3 KO rats. (**C**,**D**) Patch-clamp method shows averaged amplitude of P2X3 currents in Dil-labelled DRG neurons of WT rats and ASIC3 KO rats. (**C**): transient and (**D**): sustained P2X3 currents. An amount of 10 µM of αβ-me ATP was applied to induce current response. * *p* < 0.05 between control and occlusion in WT rats, but no significant difference in current response was seen between control and occlusion in ASIC3 KO rats. Data presented as mean ± SD.

#### 4.1.1. ASIC3 KO Suppresses the Exercise Pressor Response under Ischemic Situation

ASICs are members of a family of amiloride-sensitive sodium channels and are considered as molecular sensors in afferent neurons [90,91,92,93]. They are almost ubiquitous in the mammalian nervous system and are activated as pH drops below 7.0. Among six different proteins of ASICs (ASIC1a, 1b, 2a, 2b, 3, and 4), encoded by four genes (ASIC1, 2, 3, and 4), the ASIC3 protein, however, is mostly found in DRG where it forms functional channels that are responsive to proton concentration fluctuation [90,91,92,93]. The pH range required to activate ASIC3 is approximately 6.5–7.0 [94,95], which is close to what is observed in exercising muscle and/or moderately ischemic tissues [96,97,98,99].

Prior to utilizing the genetic approach of ASIC3 knockout, several studies were performed to assess the role played by ASIC3 in evoking the exercise pressor reflex in PAD by comparing with sham control rats on the protein expression currents [82] of ASIC3 in DRG [81], and the blood pressure responses before and after the application of pharmacological blockades (e.g., amiloride and APETx2) [100]. ASIC3 knockout provides a powerful tool to validate the role of ASIC3 on the exercise pressor reflex in PAD as it not only blocks the function of this receptor but also induces a significant reduction of the protein expression. 

In the ASIC3 KO rats, the peak mean arterial pressure (MAP) and the blood pressure index (BPI) following the static muscle contraction were similar to the wide type (WT) rats when the blood flow was freely perfused. However, under the ischemia conditions induced by femoral artery occlusion, the peak MAP and BPI were significantly lower in ASIC3 KO rats than in the WT rats. This effect was not seen in the EPR response induced by the passive tendon stretch. Researchers also injected the solutions of diprotonated phosphate (86 mM; pH 6.0), lactic acid (12 mM; pH 2.85), and capsaicin (0.2 μg; pH 7.2) to evoke the EPR response. Compared with the WT rats, the EPR response induced by diprotonated phosphate and lactic acid was significantly attenuated in ASIC3 KO rats. Interestingly, the EPR response induced by capsaicin (0.2 μg; pH 7.2) was also attenuated. However, blocking the ASIC3 in ligated WT rats by APETx2 did not suppress this capsaicin induced EPR response. This suggests there may be a special interaction or coupling effect between ASIC3 and TRPV1 receptors during the activation of TRPV1. 

#### 4.1.2. ASIC3 KO Attenuates the Exercise Pressor Response and the Activities of P2X3 under Ischemic Situation

Apart from the potential interaction between ASIC3 and TRPV1, it has been reported that there is a functional interaction between ASIC3 and P2X3 receptors [93,101]. In a published work, we used ASIC3 KO rats to examine the underlying mechanisms by which ASIC3 receptors affect P2X3 functions in regulating the EPR following femoral artery occlusion. Figure 6 shows that compared with wild-type (WT), ASIC3 KO attenuated the exaggeration of the BP response to injections of α,β-me ATP, a P2X3 agonist, into the arterial blood supply of the hindlimb muscles of occluded rats. This result is consistent with the notion suggested by our previous work that blocking ASIC3 signaling pathways can attenuate amplification of the BP response to stimulation of P2X3 receptors under the acidic milieu of the hindlimb muscles [102].

We further determined if ASIC3 KO attenuates P2X3 currents in DRG neurons innervating ischemic muscles. Figure 6 shows that muscle DRG neurons from both WT rats and ASIC3 KO rats exhibited the typical transient and sustained current responses with activation of P2X3 receptors by applying α,β-me ATP. The data further show that femoral artery occlusion augmented the amplitude of P2X3 currents in response to α,β-me ATP in muscle DRG neurons of WT rats, but this effect appeared to be less in ASIC3 KO rats. This result further supports the notion that inhibition of ASIC3 has a regulatory role in P2X3 function, and this is likely to be involved in causing the exaggerated EPR in PAD rats following femoral artery occlusion.

### 4.2. Other Ischemia-Induced Products

In addition to ASIC3, TRPV1, and P2X3, it must be pointed out that other muscle afferents’ receptors, including µ-opioid and thromboxane (TP) receptors, etc., are engaged in processing chronic ischemia of the hindlimb muscles [84,85]. In addition, studies showed that bradykinin B2 and peripheral δ-opioid receptors contribute to the exaggerated exercise pressor reflex via a mechanically sensitive group III muscle afferents in rats with femoral artery occlusion [87,103]. Blocking PGE2 EP4 also attenuates the augmented BP response to static exercise observed in PAD rats [86]. Meanwhile, the role of nerve growth factor (NGF) in regulating the metabolic receptors in the ischemia-muscles of PAD has been extensively discussed in one of the previous reviews [104], highlighting its elevation under the ischemia condition and upregulating the protein expression and function of ASIC3, P2X3, and TRPV1 in the DRG neurons. In this review, we will focus on extending our discussion on the role of hypoxia-inducible factor 1α (HIF-1α) and the reactive oxygen species (ROS). 

#### 4.2.1. HIF-1α

HIF-1 is a heterodimeric protein composed of constitutively expressed HIF-1α and HIF-1β subunits [105]. In the two subunits, oxygen-sensitive HIF-1α accumulates rapidly under hypoxic conditions and modulates the expression of several target genes in protecting tissues against ischemia and infarction [106,107,108,109]. HIF-1α is considered a transcription factor that mediates adaptive responses to hypoxia and ischemia [106,107,108,109]. Thus, we have examined if arterial occlusion increases the levels of HIF-1α in sensory neurons and if engagement of HIF-1α is responsible for the enhancement in the reflex cardiovascular responses induced by activation of muscle afferent nerves [110].

The first insight we gained in this previous study by using western blot analysis showed that HIF-1α protein expression is significantly increased in DRG neurons 6–72 h after femoral artery ligation as compared with non-ligated controls [110]. This result suggests that femoral occlusion induces HIF-1α response in sensory nerves. In addition, DMOG, an inhibitor of prolyl hydroxylase, has been shown to stabilize or increase HIF-1α protein and enhance the expression of downstream target genes [111,112]. It was reported that inhibition of endogenous HIF inactivation by DMOG induces angiogenesis in the ischemic skeletal muscles of mice [112]. In this previous study, we further examined the expression of HIF-1α protein in DRG neurons induced by intramuscular injection of DMOG [110]. HIF-1α protein expression was significantly increased in lumbar DRG neurons 24 h after injection of DMOG into the hindlimb muscles as compared with sham controls. In this prior report, we also examined the effects of femoral occlusion on the reflex cardiovascular responses evoked by activation of muscle afferent nerves [110]. Our data have shown that 24 h of femoral artery occlusion significantly increased arterial BP response induced by static muscle contraction. To determine if HIF-1α has a potential effect on the exercise pressor reflex, we injected DMOG into the hindlimb muscles. Then, BP and HR responses induced by static muscle contraction were examined 24 hrs after DMOG injection. Our results showed that there were no significant differences in increases of the reflex MAP and HR responses after DMOG as compared with controls [110].

In contrast, BAY87, a synthesized compound with characteristics of highly potent and specific suppressive effects on expression and activity of HIF-1α, was given into the arterial blood supply of the ischemic hindlimb muscles three hours before the exercise pressor reflex was evoked by static muscle contraction. First, arterial injection of BAY87 inhibited expression of HIF-1α in the DRG of occluded limbs three hours following its injection. Second, muscle contraction evoked a greater increase in BP in occluded rats and BAY87 attenuated the enhanced BP response in occluded rats to a greater degree than in control rats. Taken together, these data suggest that inhibition of HIF-1α alleviates exaggeration of the exercise pressor reflex in rats under ischemic circumstances of the hindlimbs in PAD induced by femoral artery occlusion; however, an increase in HIF-1α of DRG neurons per se may not alter the muscle pressor reflex.

Nonetheless, it should be noted that the time courses are very similar in increased HIF-1α expression, and elevated NGF and amplitude of DRG response to stimulation of ASIC3, P2X3, and TRPV1receptors after ischemic insult induced by the femoral artery occlusion [83,113,114,115]. This similarity may indicate that there is a close relationship between NGF and HIF-1α responses in the DRG neurons in the processing of muscle ischemia. Interestingly, published work shows that increasing HIF-1α or inhibiting HIF-1α prolyl hydroxylases can attenuate NGF deprivation-induced effects on neurons, suggesting that HIF-1α plays a regulatory role in affecting effects of NGF [116,117,118]. Therefore, we postulate that HIF-1α likely contributes to the effects of NGF on augmented muscle metabolic responses in the DRG neurons after arterial occlusion.

#### 4.2.2. Reactive Oxidative Species

Notably, a number of studies suggest that reactive oxidative species (ROS) contribute to the regulation of discharges of vagal lung thin afferent fiber nerves [119,120]. Additionally, it has been reported that an increase in muscle NADPH oxidase-derived ROS sensitizes the exercise pressor reflex in a decerebrate rat model [121]. Likewise, a decrease in ROS can attenuate the reflex [121]. Thus, it is speculated that ROS is engaged in augmented SNA and BP response during activation of the exercise pressor reflex in rats with femoral occlusion. Superoxide dismutases (SOD), are a class of enzymes that catalyze the dismutation of superoxide into oxygen and hydrogen peroxide as considered an important antioxidant. In a published work, tempol, a mimic of SOD, was arterially injected into the hindlimb muscles of rats and results demonstrated that tempol attenuates BP response evoked by contraction of occluded hindlimb muscles, but the attenuation was not seen when contraction was induced in freely perfused control legs [122]. A following study suggested that effects of tempol on the BP response during contraction are via ATP-dependent potassium channels [123]. However, a prior study suggested that ROS plays an important role in regulating discharges of vagal lung thin afferent fiber nerves via engagement of TRPV1 and P2X receptors [119,120]. In those experiments, the reflex pulmonary chemical response induced by a ROS stimulant hydrogen peroxide was attenuated by the prior application of i-RTX (TRPV1 antagonist) and PPADS (P2X antagonist) [119,120]. Thus, it is likely that ROS can alter the response of sensory nerves with activation of TRPV1 and P2X. Nevertheless, the augmented exercise pressor reflex is significantly attenuated after tempol is given to compensate SOD in occluded muscles of rats [122]. 

In addition, ROS activates the transient receptor potential channel A1 (TRPA1) [124,125,126]. TPRA1 is a member of branch A of the transient receptor potential (TRP) family of nonselective cation channels and expressed in the sensory (nerves) neurons and is involved in acute and inflammatory pain [124,127,128,129,130,131,132]. A published work has demonstrated that intra-arterial injection of AITC, a TRPA1 agonist, into the hindlimb muscle circulation of healthy rats led to increases in SNA and BP via a reflex mechanism [133]. Additionally, this study has suggested that TRPA1 plays a role in regulating the exercise pressor reflex and acid phosphate, bradykinin, and arachidonic acid, which are accumulated in exercising muscles are likely engaged in the role played by TRPA1 as endogenous stimuli. Interestingly, it was observed that femoral artery occlusion (1) upregulates the protein levels of TRPA1 in DRG tissues; (2) selectively increases expression of TRPA1 in DRG neurons supplying metabolically sensitive afferent nerves of C-fiber (group IV); (3) enhances renal SNA and BP responses to AITC (a TRPA1 agonist) injected into the hindlimb muscles, and (4) blocks TRPA1 attenuates SNA and BP responses during muscle contraction to a greater degree in ligated rats than those responses in control rats. Overall, the results of these studies indicate that alternations in muscle afferent nerves’ TRPA1 likely contribute to the enhanced sympathetic and BP responses via the metabolic component of the muscle reflex under circumstances of chronic muscle ischemia in PAD, and the effects of oxidative stress are also likely associated with expression and activities of TRPA1 in sensory nerves of PAD.

#### 4.2.3. Endothelin-1 (ET-1)

ET-1 is originally characterized as an endothelium-derived peptide which majorly functions as constricting factor in the vasculature [134,135]. It affects several tissues including the smooth muscle and the nervous system [136]. During inflammatory conditions, ET-1 has been found to be associated with an inflammatory response involving the expression of proinflammatory cytokines including TNF-α, IL-1 and IL-6 [137]. Meanwhile, ROS stimulates the production of ET-1 in both in vivo and in vitro situations [138,139]. In PAD patients, it has been reported that the ET-1 was elevated in the plasma [140,141]. In an animal model of PAD, the ET-1 concentration was also elevated in the gastrocnemius muscle [142]. Of note, ET-1 is an important vasoconstrictor for the restraint of blood flow in active skeletal muscle and the maintenance of arterial BP during exercise [143]. The underlying mechanism of the ET-1 on EPR response in PAD patients has not yet been fully investigated. However, a number of previous studies of the peripheral nerve establish a fundamental rationale for further mechanism and intervention studies on the ET-1-related cellular and molecular pathways during evoking the EPR response in PAD. 

In the peripheral nervous system, two subtypes of ET-1 receptors, ETᴀ and ETв, are expressed in nociceptive primary sensory neurons such as the DRG neurons, whereas ETв is mainly found in satellite glial cells [144]. Numerous studies have demonstrated that ET-1 plays an important role in modifying peripheral pain signaling. In humans, exogenous ET-1 causes tactile allodynia and severe pain. In rodents, an intra-plantar injection of ET-1 produces mechanical and thermal hyperalgesia and spontaneous pain-like behaviors [145,146]. Paw withdrawal thresholds to mechanical stimuli and heat are significantly altered in conditioned ET-1 knockout mice [147]. Peripheral ET-1 acts on nociceptors through its cognate receptors, which subsequently modify pain-related ion channels to amplify signal generation [148]. In DRG neurons, ET-1 increases neuronal excitation by hyperpolarizing tetrodotoxin-resistant (TTX-R) Na+ channels and by suppressing the delayed outward rectifier K+ current via ETᴀ receptor [149,150]. In contrast, ET-1 can decrease the excitability of DRG neurons through the activation of ETв receptors [151]. It has been also reported that ETᴀ and ETв receptors play a role in regulating sensitivity of the peripheral sensory nerve in neuropathic pain induced by spinal nerve ligation in rats [152]. 

### 4.3. Pro-Inflammation Cytokines and Ion Channels in Muscle Sensory Neurons (Figure 7 and Figure 8)

#### 4.3.1. TNF-α and Activities of Nav Channels in Muscle DRG Neurons

The augmented exercise pressor reflex might be due in part to inflammation, specifically pro-inflammatory cytokines (PICs) associated with PAD. Numerous cells (i.e., leukocytes, myocytes, microglia, astrocytes, and Schwann cells) produce and release PICs [153], which include interleukins, lymphokines, and cell signaling molecules. In particular, the roles of tumor necrosis factor-α (TNF-α), interleukin-6 (IL-6), and interleukin-1β (IL-1β) are significant in regulating immune and inflammatory reactions. These PICs modulate the activities of many cell types in various diseases. For example, during diseased states, PICs help to recruit cells to inflammatory sites, stimulating cell survival, division, and enhancing proliferation and differentiation [154]. Evidence indicates that PICs are involved in regulating physiological functions, with their levels increasing in the circulation and in the affected tissues [153,155,156]. Increased circulating and intramuscular levels of PICs (such as IL-6 and TNF-α) were also found in coronary and/or atherosclerotic vascular disorders such as PAD [157,158,159]. 

**Figure 7:**
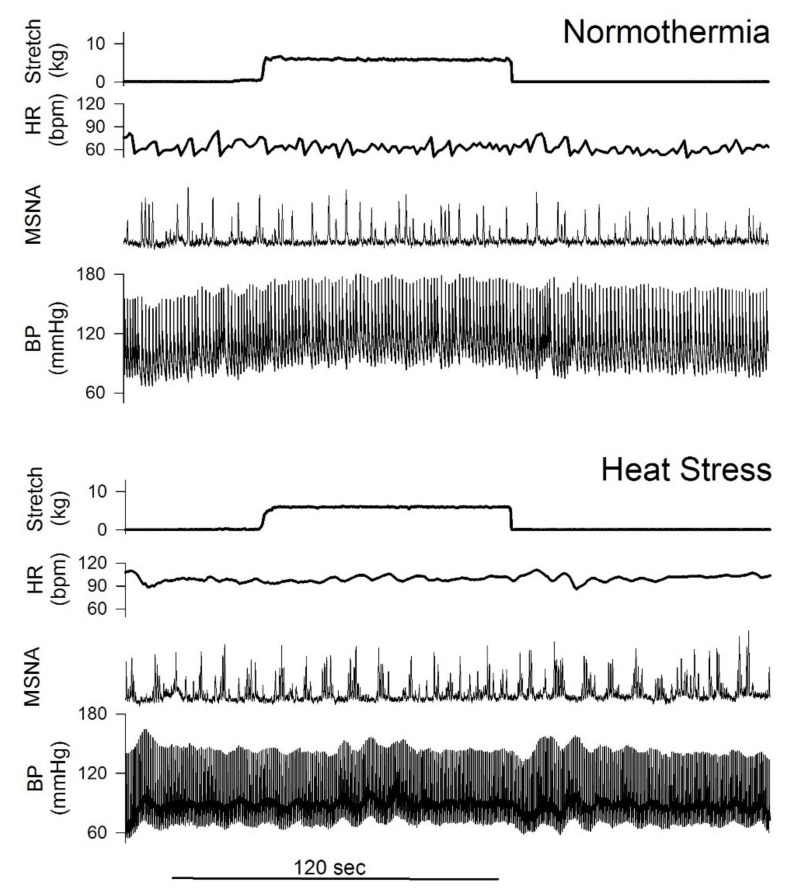
Passive stretch during post-exercise circulatory occlusion evoked MSNA and BP increases in normothermic conditions. These responses were attenuated under heat stress conditions. (Unpublished figure).

**Figure 8:**
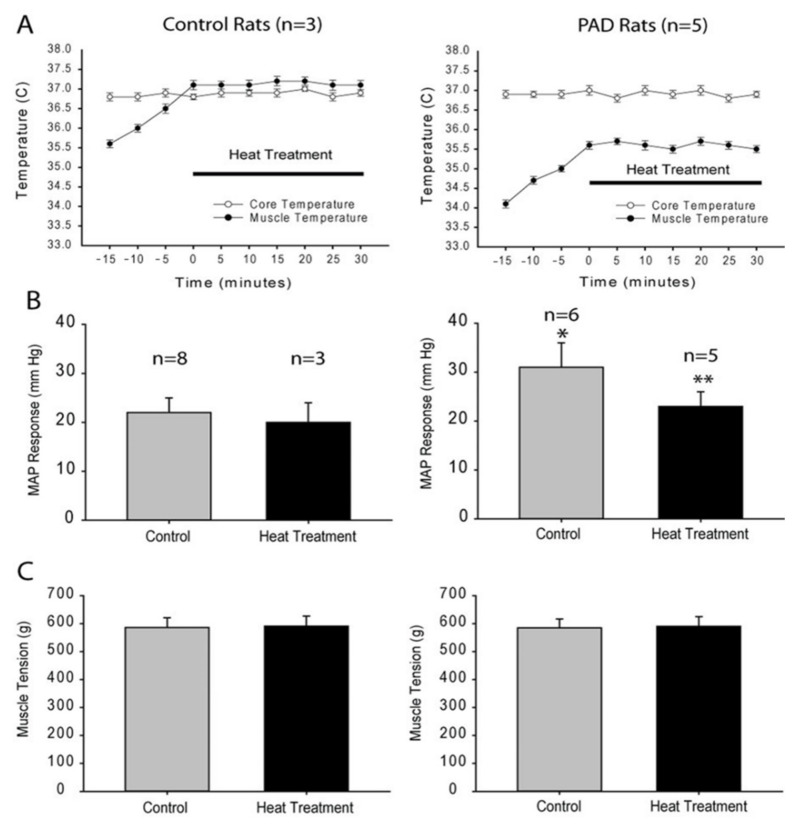
Tcore and Tm, MAP response and muscle tension in control rats (left panels) and PAD rats (right panels). (**A**): Baseline Tm was lower in PAD rats and Tcore was not altered during heat treatment. (**B**): MAP response to contraction was increased in PAD rats and amplification of pressor response was attenuated after heat treatment. * *p* < 0.05 vs. control rats; and ** *p* < 0.05 vs. control group without heat treatment. (**C**): No difference in muscle tension among groups (*p* > 0.05).

It was first observed that the levels of TNF-α and protein expression of TNF-α receptor type 1 (TNFR1) were increased in the DRG of the hindlimbs of PAD rats. Note that TNF-α was observed within DRG neurons of C-fiber afferent nerves. Capsaicin (TRPV1 agonist) and AITC (TRPA1 agonist) were injected into the arterial blood supply of the hindlimbs to stimulate metabolically sensitive thin-fiber muscle afferents. The effects of these injections on the SNA and pressor responses were attenuated in PAD rats after TNF-α synthesis suppressor pentoxifylline (PTX) was previously administered into the hindlimb with femoral artery occlusion. These data suggest that TNF-α plays a role in modulating exaggerated SNA via the metabolic component of the exercise pressor reflex in PAD. 

Tetrodotoxin (TTX)-resistant Na^+^ (i.e., Na_V_1.8) channels are highly expressed in group IV afferents [160]. The role played by Na_V_1.8 in evoking the exercise pressor reflex was examined using whole animal preparations. A803467, a Na_V_1.8 blocker, attenuates the pressor response evoked by arterial injection of lactic acid and capsaicin stimulating thin fiber afferents [161]. There is a linkage between TNF-α and the activity of Na^+^ current in sensory nerves [162]. A prior study demonstrated the role of TNF-α in enhancing the current densities of Nav1.8 in DRG neurons [163]. In an additional work, the role played by TNF-α in regulating the activity of NaV1.8 currents in muscle DRG neurons of PAD rats was specifically examined. Results showed that peak amplitude of TTX-resistant (TTX-R) Nav and NaV1.8 currents in muscle DRG neurons were increased in PAD rats. Meanwhile, the amplification of TTX-R and NaV1.8 currents induced by TNF-α was attenuated in DRG neurons with pre-incubation with respective inhibitors of the intracellular signaling pathwaysp38-MAPK, JNK, and ERK. It was concluded that NaV1.8 is engaged in the role of TNF-α in amplifying muscle afferent inputs as the hindlimb muscles are ischemic in PAD. The pathways of p38-MAPK, JNK, and ERK are likely necessary to mediate the effects of TNF-α. 

#### 4.3.2. IL-6 and Activities of Kv4 Channels in Muscle DRG Neurons

Increased circulating and intramuscular levels of interleukin-6 (IL-6) are detected in PAD patients [164,165]. The activity of exercise induces a greater increase in the levels of IL-6 of the mixed venous blood in PAD patients than those levels in healthy age-matched subjects [166,167]. Consistently, during the exercise ischemic insult also enhances the circulating IL-6 levels compared with non-ischemic exercise [168].

Seventy-two hours of femoral artery occlusion increases products of oxidative stress in the hindlimb muscles of rats and activates inflammatory signaling pathways [74,75,76]. IL-6 also plays a role in regulating the exaggerated BP response to static exercise in PAD rats [169] likely via membrane-bound IL-6R or gp130 trans-signaling pathways assembled by soluble forms of IL-6R [163,170,171]. Thus, it was anticipated that the activity of IL-6 signaling would be increased in muscle afferent nerves involving the exercise pressor reflex in PAD rats.

We found that the protein levels of IL-6 and its receptor IL-6R expression were increased in the DRGs of PAD rats with 72 h of femoral artery occlusion. Inhibition of muscle afferents’ IL-6 trans-signaling pathway (gp130) by intra-arterial administration of SC144, a gp130 inhibitor, into the hindlimb muscles of PAD rats alleviated BP to static muscle contraction. On the other hand, it was found that PAD decreased amplitude of Kv4 currents in rat muscle DRG neurons. The homo IL-6/IL-6Rα fusion protein (H. IL-6/6Rα) but not IL-6 alone significantly inhibited Kv4 currents in muscle DRG neurons; the effect of H. IL-6/6Rα was largely reverted by SC144. Consistent with the previous findings, these data suggest that via trans-signaling pathway upregulated IL-6 in muscle afferent nerves by ischemic hindlimb muscles inhibits the activity of Kv4 channels and therefore likely leads to adjustments of the exercise pressor reflex in PAD. 

## 5. Heat Treatment and Nutrition Intervention on Improving Exercise-Induced Exaggerated SNA and BP Responses in PAD

Supervised exercise intervention is one of the most effective means of maintaining or restoring the exercise tolerance of PAD patients [12,172]. However, as above mentions, the challenge exists in the adherence to exercise training programs due to the symptom of intermittent claudication, which is partly attributed to the exaggerated EPR response. Therefore, in this review, we discuss three other non-pharmacological interventions that may be helpful to ameliorate the hyper-amplified EPR response in PAD patients. With the introduction of those promising economic strategies, we are aiming to incorporate them into the well-established exercise training protocols to enhance the adherence to exercise training, improve the efficacy of the intervention protocols, and benefit the overall well-being of PAD patients. 

### 5.1. Heat Treatment (Figure 7 and Figure 8)

In recent years, heat treatment has been obtaining significant attention in terms of its beneficial effect on cardiovascular patients including PAD. In a rat model of PAD, the heat treatment protocol of increasing the muscle temperature (Tm) by 1.5 °C (30 min period each heating protocol, 2 times/day for 3 days) attenuated ET-1 in both red and white portions of gastrocnemius muscle in PAD [142]. It has been reported that repeated heating exposure suppressed the production of plasma ET-1 in human participants with symptomatic PAD in both rest [173] and post-exercise situations [174]. Meanwhile, heating exposure increases the contraction force of the ischemia-induced damaged skeletal muscle [175]. In a mice obesity model, the heating exposure also decreased the percentage of fat and increase the ratio of muscle mass to body mass even if capillary density and collateral supply diameter was unchanged [176]. 

In terms of the blood flow dynamic response, heat exposure increases skin blood flow (SkBF), heart rate, cardiac output [177], ejection fraction, and systolic function in healthy individuals [178,179] and heart failure patients [180,181]. Based on these observations, thermal therapy has been suggested for patients with heart diseases [180,181,182,183,184]. Moreover, it has been shown that heat treatment (e.g., dry sauna) may improve chronic endothelial function in patients with heart diseases or in those with atherosclerotic risk factors [183,185,186,187]. A recent report also shows that hot water immersion raised the blood flow in lower limbs of PAD patients [188]. It should be noted that these prior studies [188,189,190] only focused on the effects of heating on blood flow and vascular function in PAD. The effects of heat exposure on EPR in PAD have not been examined. 

Thus, the effects of heating on EPR were studied in PAD patients and PAD rats induced by femoral artery occlusion. First, to determine how whole-body heating alters muscle mechanoreflex and metaboreflex responses, we measured MSNA in healthy subjects during fatiguing isometric handgrip exercise, PECO, and passive muscle stretch (extension of wrist, EOW) during PECO. The protocol was performed under both normothermic and whole-body heating (ΔTcore ~0.6 °C via a heating suit) conditions. Under normothermic conditions, passive stretches during PECO evoked significant increases in mean arterial pressure (MAP) and MSNA. However, during heating, passive stretch did not significantly increase MSNA or MAP (Figure 7). These data show that sympathetic response to the mechanoreceptor stimulation [191] is attenuated by heat exposure when body temperature is elevated [192]. The attenuated MSNA response to stretch during heating should not be a “ceiling effect” because there was no significant difference in the MSNA burst incidence during stretches between thermal conditions. 

Although there was no difference in MSNA response to PECO (i.e., non-specific metaboreceptor stimulation), the MAP response to PECO during heating was much less (by ~50%) than in normothermic conditions. Thus, the EPR (i.e., pressor response) is attenuated during heating. It is speculated that the BP response to sympathetic activation is also attenuated during heating [193]. It is known that MSNA response to metaboreceptor stimulation is attenuated in heart failure [194]. On the other hand, it is unclear if the MSNA response to metaboreceptor stimulation is altered in PAD. Therefore, it is necessary to examine both MSNA and BP responses to exercise in PAD. 

A prior study [195] demonstrated that local heating of an isometric exercising forearm muscle group augmented the increase in MSNA during fatiguing exercise. They speculated that the elevated Tm might sensitize muscle mechanosensitive afferents. It should be noted that in those studies, local heating increased forearm Tm from ~34 to 39 °C [195,196,197]. Our pilot study shows that whole-body heating only raised forearm Tm of ~1.5 °C. Thus, the fewer increases in the Tm in our studies are likely to lead to the different effects on the muscle afferents at the receptor level, necessitating the study engagement of P2X in the EPR after heat exposure. 

Group III and IV respond to changes in Tm [198,199]. In animals, we have shown that a higher Tm response is linked to a lower BP response and elevated Tm attenuates the P2X receptor-mediated reflex activation of muscle mechano- and metabo-receptors [200]. We have shown that arterial injection of α,β-me ATP into the hindlimb muscles evoked a dose-dependent response, and the peak pressor response evoked by α,β-me ATP was attenuated as Tm was increased by heat exposure. Additionally, α,β me ATP amplified the reflex BP response evoked by stretch and the effect was blunted with heat exposure. 

The effects of heat exposure on EPR in PAD have not been well understood. Due to limb ischemia, the lower limb temperature is lower and revascularization therapy raised the limb temperature [201]. In addition, a decrease in the Tm induces a decelerated rate of ATP turnover [202], which likely leads to an elevation of ATP concentration in the extracellular space. It is, therefore, speculated that the lower Tm may contribute to the accentuated EPR in PAD. In turn, a suitable rise in Tm may attenuate the sympathetic response and decrease the exaggerated BP response to exercise in PAD. To obtain the same degree of Tm increase in animal models, the temperature in rat hindlimb muscle was monitored and increased by 1.5 °C (30 min period each heating protocol, two times/day for three days) and then BP response to static muscle contraction was examined (Figure 8). These data demonstrated that a raise in muscle temperature attenuated the exaggerated BP response to muscle contraction. This study further showed that a protocol with increasing muscle temperature by 1.5 °C decreased expression and current response of P2X3 in DRG neurons of PAD rats, suggesting P2X3 signaling is a part of the mechanisms leading to inhibition of BP response. Based on those published results in humans and animals, it is speculated that heat exposure and/or heat treatment would be beneficial to attenuate the sympathetic response and decrease the exaggerated BP response in PAD. 

The effect of a short period heating intervention on relieving the symptoms of intermittent claudication is also intriguing to study. In one of the previous studies, the one-time acute effect on the EPR response in PAD was evaluated. The muscle temperature was increased by 1.5 °C and the length was 5 min. The EPR response induced by both static muscle contraction and α,β-Me-ATP injection was evaluated 20 min before, immediately after, and 20 min after the heat exposure. The results of this study were interesting as the static muscle contraction induced EPR was attenuated following the heat exposure, and the EPR response attenuation was recovered 20 min after the muscle temperature returned to the baseline. However, the α,β-Me-ATP-induced EPR response did not alter with heat exposure. This suggested the attenuation of static-muscle-contraction-induced EPR response following one-time heat exposure may not work through alternating the expression and function of P2X3 receptors. Instead, it may work through the alternation of the ATP metabolism enzyme activity, e.g., ATPase, which will be one of the further directions of the mechanism study on this topic. As intermittent claudication frequently occurs and interrupts the daily physical movement of the patients, this study provides a fundamental basis for the daily base intervention strategy for the PAD patients.

### 5.2. Effects of Supplemental Nutrients

#### 5.2.1. Vitamin B6

A diet deficient in vitamin B6 leads to a decreased activity of cystathionine β-synthase and cystathionase in the liver. Dietary supplementation of vitamin B6 stimulates the activity of these enzymes and increases the endogenous synthesis of cysteine from methionine. In hypertensive animals and humans, increased production of cysteine would lead to more efficient excretion of excess metabolic aldehydes, normalizing vascular calcium channels and lowering blood pressure [203]. A regression study has also shown that an increase in the daily intake of vitamin B6 by one standard deviation (approximately 0.5 mg per day) would reduce the risk of PAD by 29% [204]. More importantly, once consumed vitamin B6 will be converted into a P2-purinoceptor antagonist called pyridoxal-5-phosphate (PLP) [205]. In animal studies, the intraperitoneal injection of the vitamin B complex (B1/B6/B12 = 100/100/2 mg/kg) attenuated the expression of P2X3 in DRG of diabetic rats [206]. By locally infusing the vitamin B6 into human participants’ forearms, previous studies [207] suggested that the MSNA responses to fatiguing handgrip, post-exercise circulatory occlusion (PECO), and PECO + passive stretch were all significantly less than those before pyridoxine. The blood pressure responses were also significantly less than those before vitamin B6 infusion.

#### 5.2.2. Vitamin C

Low levels of Vitamin C supplementation (assessed by dietary intake or plasma analysis) are associated with multiple conditions, including high blood pressure (BP), endothelial dysfunction, heart disease, atherosclerosis, and stroke [208]. For the mechanism work on the Vitamin C supplementation on blood pressure regulation, the information is lacking in terms of the efficacy of Vitamin C supplementation on the ERP response in cardiovascular patients, especially in PAD, and the IR injury of PAD patients following the revascularization surgery. A previously performed human study [17], investigated the efficacy of Vitamin C intravenous infusion in attenuating oxidative stress and therefore the subsequent EPR responses in PAD patients. In this study, the Vitamin C infusion elicited a lower MAP response to low-intensity rhythmic plantar flexion in the affected legs of PAD patients than that in the condition without Vitamin C infusion. 

## 6. Conclusions

Studies using a rat model of femoral artery occlusion show that sympathetic responses of the exercise pressor reflex engagement are exaggerated as observed in PAD patients. As summarized in Figure 1, findings of the completed studies suggest that enhanced protein levels of ASIC3, P2X3, and TRPV1 in muscle afferent nerves and amplified responses of those receptors contribute to the exaggerated reflexive sympathetic and pressor responses to their individual receptor stimulus. The findings further suggest that NGF is likely responsible for enhanced ASIC3, P2X3, and TRPV1 and plays a role in modulating the metaboreceptor component of the exercise pressor reflex in hindlimb muscle ischemia. Lactic acid, ATP, and acid phosphate are the major muscle by-products in exercising muscles and ASIC3, P2X3, and TRPV1 receptors are sensitive to those individual metabolites and/or combined metabolites. Overall data presented here provide evidence that alteration in chemically sensitive receptors ASIC3, P2X3, and TRPV1 in primary afferent neurons innervating ischemic muscles plays an important role in the development of the exaggerated reflexive sympathetic responses, likely leading to worsening exercise capacity in patients with PAD. Moreover, NGF in sensory nerves plays a role in regulating abnormal responses of those metabolic receptors. Also, HIF-1α likely contributes to the effects of NGF on augmented muscle metabolic responses in the DRG neurons after arterial occlusion. A study limitation needs to be mentioned based on the fact that the studies included in this current review are varied from factors such as sample size, gender ratio, and the choices of different animal models as well as human populations. Therefore, additional studies are warranted to verify and confirm the underlying mechanism and clinical results. More animal models to exemplify different stages or pathological conditions of PAD are also necessary to explore. In combination with the fundamental work performed by the previous studies, those mechanistic foundations formed by studies in animal models will shed light on the targets of the translational intervention studies to alleviate the adverse effects that increase the cardiovascular event risk in PAD patients.
